# Supplementary material for: Offering ART refill through community health workers versus clinic-based follow-up after home-based same-day ART initiation in rural Lesotho: The VIBRA cluster-randomized clinical trial
Source: PLoS Med. 2021 Oct 21;18(10):e1003839. doi: 10.1371/journal.pmed.1003839 (PMC8568187; doi:10.1371/journal.pmed.1003839)
Supplement: S1 Table — (DOCX) [file pmed.1003839.s003.docx]

**S1 Table:** Sensitivity analyses on primary endpoints

|  | **Total (N=257)** | **Control (n=139)** | **Inter-vention (n=118)** | **Odds ratio**  **(95% CI) [1,3]** | **Risk ratio (95% CI) [1,2,3]** | **Risk difference**  **(95% CI) [1,2]** | **P-value [1]** |
| --- | --- | --- | --- | --- | --- | --- | --- |
| ***Sensitivity analyses on primary endpoints*** |  |  |  |  |  |  |  |
| Sensitivity: VLs up to 30 days past protocol-defined window [4] |  |  |  |  |  |  |  |
| VL <20 copies/ml | 112 (44%) | 65 (47%) | 47 (40%) | 0.74 (0.44 to 1.22) | 0.84 (0.60 to 1.08) | -0.07 (-0.20 to 0.05) | 0.235 |
| VL <400 copies/ml | 137 (53%) | 78 (56%) | 59 (50%) | 0.75 (0.46 to 1.24) | 0.88 (0.67 to 1.08) | -0.07 (-0.19 to 0.05) | 0.267 |
| Sensitivity: individual per protocol set [5] |  |  |  |  |  |  |  |
| VL <20 copies/ml | 106 (65%) | 61 (65%) | 45 (64%) | 1.03 (0.48 to 2.22) | 1.01 (0.76 to 1.26) | 0.01 (-0.15 to 0.17) | 0.932 |
| VL <400 copies/ml | 129 (79%) | 72 (77%) | 57 (81%) | 1.34 (0.61 to 2.95) | 1.06 (0.89 to 1.23) | 0.05 (-0.08 to 0.18) | 0.464 |
| Sensitivity: Role of choosing VHW per protocol set [6] | | | | | | | |
|  | Total (n=187) | Control (n=139) | Intervention (n=48) |  |  |  |  |
| VL <20 copies/ml | 85 (45%) | 64 (46%) | 21 (44%) | 0.88 (0.45 to 1.74) | 0.93 (0.59 to 1.28) | -0.03 (-0.19 to 0.14) | 0.718 |
| VL <400 copies/ml | 101 (54%) | 75 (54%) | 26 (54%) | 0.97 (0.50 to 1.91) | 0.99 (0.68 to 1.29) | -0.01 (-0.17 to 0.16) | 0.934 |

Abbreviations: CI (confidence interval), VL (viral load), VHW (village health worker)

[1] Intervention versus control group, estimated by random effects logistic regression models.

[2] Confidence intervals estimated using delta method.

[3] Adjusted for stratification factors: district, size of village, and ease of reaching health center.

[4] Includes the results of viral loads taken 30 days past the end of the primary endpoint window of 451 days since enrolment (n=4; 3 in control group and 1 in intervention).

[5] Individual per-protocol set includes all who attended both the 6- and 12-month study visit.

[6] Compares individuals in the intervention group who chose the VHW for ART refills (n=48) to those in the control group (n=139).
