# Supplementary material for: Offering ART refill through community health workers versus clinic-based follow-up after home-based same-day ART initiation in rural Lesotho: The VIBRA cluster-randomized clinical trial
Source: PLoS Med. 2021 Oct 21;18(10):e1003839. doi: 10.1371/journal.pmed.1003839 (PMC8568187; doi:10.1371/journal.pmed.1003839)
Supplement: S2 Table — (DOCX) [file pmed.1003839.s004.docx]

**S2 Table:** Primary outcome: effect modification and subgroup analyses

|  | **VL <20 copies/mL at 12 months** | | | **Odds ratio (95% CI) [1]** | **Risk diff (95% CI) [1,2]** | **P-value** |
| --- | --- | --- | --- | --- | --- | --- |
|  | **Total (N=257)** | **Control (n=139)** | **Intervention (n=118)** |  |  |  |
| Age groups |  |  |  |  |  | 0.71 [3] |
| 10-19 | 4 | 1/2 (50%) | 1/2 (50%) |  |  |  |
| 20-24 | 24 | 1/10 (10%) | 4/14 (29%) |  |  |  |
| >24 | 229 | 62/127 (49%) | 41/102 (40%) |  |  |  |
| Gender |  |  |  |  |  | 0.51 [3] |
| Male | 107 | 23/56 (41%) | 15/51 (29%) |  |  |  |
| Female | 150 | 41/83 (49%) | 31/67 (46%) |  |  |  |
| Education |  |  |  |  |  | 0.1 [3] |
| Primary only | 205 | 56/113 (50%) | 35/92 (38%) | 0.62 (0.34 to 1.13) | -0.11 (-0.26 to 0.03) | 0.117 |
| Secondary/higher | 51 | 8/18 (31%) | 11/25 (44%) | 1.51 (0.45 to 5.09) | 0.09 (-0.18 to 0.36) | 0.505 |
| Employment |  |  |  |  |  | 0.17 [3] |
| Regular employment | 40 | 13/30 (43%) | 6/10 (60%) |  |  |  |
| No regular employment | 217 | 51/109 (47%) | 40/108 (37%) |  |  |  |
| CD4 cell count |  |  |  |  |  | 0.27 [3] |
| <350 | 74 | 17/44 (39%) | 14/30 (47%) |  |  |  |
| ≥350 | 108 | 27/55 (49%) | 21/53 (40%) |  |  |  |
| TB status |  |  |  |  |  | 0.55 [3] |
| TB suspect | 33 | 9/16 (56%) | 7/17 (41%) |  |  |  |
| No signs of TB | 221 | 55/123 (45%) | 39/98 (40%) |  |  |  |
| Excessive alcohol drinking |  |  |  |  |  | 0.52 [3] |
| Yes | 16 | 2/8 (25%) | 2/8 (25%) |  |  |  |
| No | 60 | 19/38 (50%) | 8/22 (37%) |  |  |  |
| HIV/ART history |  |  |  |  |  | 0.23 [3] |
| Newly diagnosed | 200 | 50/108 (46%) | 40/92 (43%) |  |  |  |
| Known HIV+/ART naïve | 28 | 9/17 (53%) | 4/11 (37%) |  |  |  |
| Previous ART | 29 | 5/14 (36%) | 2/15 (13%) |  |  |  |

Abbreviations: CI (confidence interval), VL (viral load), TB (tuberculosis)

[1] Stratified analysis of intervention versus control group adjusted for randomization stratification factors, estimated by logistic regression.

[2] Confidence intervals estimated using delta method

[3] P-value for interaction.
