# Supplementary material for: Offering ART refill through community health workers versus clinic-based follow-up after home-based same-day ART initiation in rural Lesotho: The VIBRA cluster-randomized clinical trial
Source: PLoS Med. 2021 Oct 21;18(10):e1003839. doi: 10.1371/journal.pmed.1003839 (PMC8568187; doi:10.1371/journal.pmed.1003839)
Supplement: S3 Table — (DOCX) [file pmed.1003839.s005.docx]

**S3 Table:** Detailed reports of the deaths occurred

| **Study ID** | **Date of enrolment** | **Date of death** | **Gender** | **Age** | **Assessment at enrolment** | **Laboratory at enrolment** | **ART and VHW** | **Follow-up** | **Overall appreviation** |
| --- | --- | --- | --- | --- | --- | --- | --- | --- | --- |
| ***V018*** | 24.09.2018 | 20.02.2019 | f | 43 | Known HIV+, ART-naïve, TB screening neg, alcohol screening neg, no clinical abnormality | - CD4: 163 cells/microL  - Hb: 5 g/dL  - Crea: 162 mcmol/l (eGFR n. CG: 25 mL/min; 40kg)  - CrAg: negative | SD-ART with ABC/3TC/EFV (no CTX); **chose VHW** | Linked to VHW, regularly initial refills through VHW. Referral to hospital due to dizziness, bloody urine, weakness. Clinical improvement at discharge. Died a month later at home. | Cause of death unclear, suggestive for renal  failure, probably acute on chronic due to a glomerulopathy. Unlikely due to ART (ABC/3TC/EFV). Immediate referral happened. IRIS possible.  Enrolment in VIBRA control would not have had a different course of events. |
| ***V022*** | 26.09.2018 | 31.10.2018 | f | 60 | Known HIV+, ART-naïve, TB screening neg, alcohol screening neg, Chronic right-sided facial paralysis, incl. loss of blinking, otherwise no further neurological or other clinical abnormalities | - CD4: 372 cells/microL  - Hb: 11.4 g/dL  - Crea: 156 mcmol/l (eGFR n. CG: 26 mL/min; 48kg) | SD-ART with AZT/3TC/EFV; **chose VHW** | Linked to VHW. No further symptoms reported during refills. Died at home. According to the grandchildren, the  participant reported tiredness and weakness on the day of death. | Cause of death unclear, according to relatives it was a sudden death, suggestive for a sudden cardiac event or a stroke. Unlikely due to ART. IRIS unlikely.  Enrolment in VIBRA control would not have had a different course of events. |
| ***V028*** | 26.11.2018 | 28.02.2019 | m | 59 | Known HIV+, ART-defaulter, TB screening neg, alcohol screening pos, clinical observation without abnormality | - CD4: 499 cells/microL  - Hb: 16.2 g/dL  - Crea: 120 mcmol/l (eGFR n. CG: 53 mL/min; 64kg) | SD-ART with TDF/3TC/EFV; **chose VHW** | Never linked to the VHW or the clinic. Several tracing attempts by the VHW and the clinic tracing team, participant refused care. Died at home. | Cause of death unclear.  Enrolment in VIBRA control would not have had a different course of events. |
| ***V097*** | 29.04.2019 | May 2019 | f | 59 | New diagnosis, alcohol screening neg, TB screening pos (weight loss; Sputum neg), no other clinical abnormalities | - CD4: 488 cells/microL  - Hb: 12.5 g/dL  - Crea: 130 mcmol/l (eGFR n. CG: 55 mL/min; 83kg) | SD-ART with TDF/3TC/EFV; **chose clinic** | Never linked to the clinic. Several tracing attempts by the clinic tracing team, participant refused care. Died at home. | Cause of death unclear.  Enrolment in VIBRA control would not have had a different course of events. |
| ***V106*** | 06.05.2019 | 01.02.2020 | m | 32 | New diagnosis, alcohol screening neg, TB screening neg, nicotine & cannabis use, no clinical abnormalities | - CD4: not available due to problems with the PIMA machine  - Hb: 13.5 g/dL  - Crea: 125 mcmol/l (eGFR n. CG: 60 mL/min; 56kg) | SD-ART with TDF/3TC/EFV; **chose clinic** | Transferred out of initial clinic. Stayed in care until August 2019, then moved place of stay again due to work. Never seen again, tracing attempts were unsuccessful. Died at home. | Cause of death unclear.  Enrolment in VIBRA control would not have had a different course of events. |
| ***V199*** | 23.10.2018 | 24.01.2019 | f | 31 | New diagnosis, alcohol screening neg, TB screening neg, no clinical abnormalities | - CD4: 263 cells/microL  - Hb: 13.5 g/dL  - Crea: 45 mcmol/l (eGFR n. CG: 141 mL/min; 56kg) | SD-ART with TDF/3TC/EFV (+CTX); **chose VHW** | Initially linked to VHW. Also visited the clinic, where she was started on IPT. Shortly after nausea, vomiting and abdominal pain. Referral to clinic, symptomatic treatment prescribed. Five days after onset of jaundice and persisting abdominal pain, referral to hospital. ART continued, CTX and IPT stopped, antibiotics administered. Elevated liver function tests including hyperbilirubinemia. After further clinical deterioration referral to higher level hospital. Died in hospital. | Cause of death unclear, probably cholestatic origin. IRIS possible. The initial elevation of Bilirubin, ALT/AST likely due to one of the newly started medications (i.e.  IPT, CTX, less likely EFV). ALT/AST significantly decreased after IPT and CTX were stopped. IRIS possible.  Immediate referrals happened. |
| ***V216*** | 15.11.2018 | 21.11.2019 | m | 56 | New diagnosis, alcohol screening neg, TB screening neg, no clinical abnormalities | - CD4: 89 cells/microL  - Hb: 17.8 g/dL  - Crea: 125 mcmol/l (eGFR n. CG: 51 mL/min; 59kg)  - CrAg: negative | SD-ART with TDF/3TC/EFV (+CTX); **chose clinic** | Linked to the clinic. Regular ART visits. 6-month VL undetectable. In October 2019 hospitalized at district hospital due to ascites. Recovered and discharged. According to relatives, shortly after complaining about abdominal pain, visited two different traditional healers, died 8 days later on the way to the hospital. | Cause of death unclear.  Enrolment in VIBRA control would not have had a different course of events. |
