# Supplementary material for: Offering ART refill through community health workers versus clinic-based follow-up after home-based same-day ART initiation in rural Lesotho: The VIBRA cluster-randomized clinical trial
Source: PLoS Med. 2021 Oct 21;18(10):e1003839. doi: 10.1371/journal.pmed.1003839 (PMC8568187; doi:10.1371/journal.pmed.1003839)
Supplement: Attachment — Submitted filename: VIBRA_response_to_reviewers_20210831.docx [file pmed.1003839.s006.docx]

**Response to Reviewers**

*Offering ART refill through community health workers versus clinic-based follow-up after home-based same-day ART initiation in rural Lesotho: The VIBRA cluster-randomised clinical trial*

***** Requests from the editors:**

1) So as to comply with PLOS' data policy, you will need to arrange a mechanism which does not involve contacting article authors for readers wishing to inquire about data access (<https://scanmail.trustwave.com/?c=6967&d=vpKD4YsEaBmPruTRBUHDweoCvxjLt9KAfs-lnzu-2Q&u=https%3a%2f%2fjournals%2eplos%2eorg%2fplosmedicine%2fs%2fdata-availability%29>)

***Response:*** *We updated our data sharing statement:*

“A key pseudo-anonymized individual participant dataset collected during the study, along with a data dictionary, will be made available at the time of publication through the data repository Zenodo with open access.”

*As soon as the DOI is available, we will forward it to you.*

2) Please add a new final sentence to the "Methods and findings" subsection of your abstract, which should begin "Study limitations include ..." or similar and should quote 2-3 of the study's main limitations. 
***Response:*** *We adapted accordingly:*

“Study limitations include a rather small sample size, 9% missing viral load measurements in the primary endpoint window and substantial migration among the study population.”

3) In the author summary, please use the active voice (e.g., "We found that ...") in at least one point.
***Response:*** *We adapted accordingly.*

4) Please rename figure 1 "Participant flowchart" or similar.

***Response:*** *We adapted accordingly.*

5) Where quoting 95% CI in which one bound is less than zero, we suggest separating the bounds with "to" so that minus signs are easily interpreted.

***Response:*** *We adapted accordingly.*

6) Please avoid claims of "the first" and the like, and where necessary add "to our knowledge" or similar.
***Response:*** *We adapted accordingly. See first sentence in “What did the researchers do and find?” of the author summary and first sentence of the last paragraph in the discussion.*

7) Throughout the text, please adapt reference call-outs to the following style: "... initiation [7].".

***Response:*** *We adapted accordingly.*

8) In the reference list, italics should be converted into plain text.

***Response:*** *We adapted accordingly.*

9) Please list 6 author names rather than 3, followed where appropriate by "et al.".

***Response:*** *We adapted accordingly.*

10) Please include a completed CONSORT checklist as a supplementary file, labelled "S1_CONSORT_Checklist" or similar and referred to as such in your Methods section. In the checklist, please refer to individual items by section (e.g., "Methods") and paragraph number, not by page or line numbers as these generally change in the event of publication.

***Response:*** *We submitted a CONSORT checklist and referred to it in the last paragraph of “Study design and participants”*

***** Comments from academic editor:**

1) The referees' comments, in different ways, really all relate to the justification for the intervention, the small numbers, the difficulty to generalise to other settings and the use of strict p-value criteria rather than interpreting the results with more insight. Overall, this looks like a major revision, to see if the next resubmission would be more insightful and helpful for the wider HIV programme community. It is the case that sustainably engaging people into HIV/ART care remains a challenge and using community-based ART is seen as a likely way forward - this makes it all the more important for this paper to put the results in their context, and to discuss how results can (or cannot) be generalised to other settings. Reviewer 3 questions whether this trial was the correct one to embark on - and I would agree that there should be more detailed justification for the trial design. For example, from the results of the previous trial in the same setting, what lessons were learnt for lack of access/engagement in ART care? In that previous trial, CASCADE, only 2/3 participants in the intervention (home-based ART initiation followed by clinic ART care) engaged in care. What were the reasons in that trial that people did not access ART care, and why did the authors decide that the solution would lie in making ART available in the community from time of initiation onwards (rather than have ART stabilised at the clinic before making refills available in the community). Was a trial with three arms contemplated? So, better justification is definitely required, and then the discussion needs to get into the possible reasons for the small difference between the arms in the current trial.

***Response:*** *Thank you for pointing out this important aspect. The VIBRA intervention was designed during a series of workshops and based on data from previous trials as well as the UNAIDS community health worker policy. We added the following paragraph in section “Intervention and control”:*

“The VIBRA intervention package was designed during a series of workshops with various stakeholders in Lesotho, including community members, clinicians and district Ministry of Health authorities. These workshops identified community-based ART refill by VHWs after home-based same-day ART initiation as a potentially promising, feasible and sustainable intervention to improve linkage and retention in care. This assumption was made based on the following reasons: First, the VHWs are rooted and largely respected in their rural community and refill at village level would minimize travel time and cost to patients. In previous studies conducted in the same setting, VHWs were identified as a trusted cadre for adults and adolescents during community-based HIV testing and counselling. Further, the two-year follow-up of participants who did not link to care after offering home-based same-day ART initiation during the CASCADE-trial revealed that a majority did not link because they did not trust or dislike the care provision at their formal health care facility. We thus hypothesized that for these persons follow-up by the VHW could be an attractive alternative.

Second, the VHWs are an already established pillar of the health system and are part of UNAIDS long-term policy, thus offering a sustainable, scalable and policy-aligned approach.”

*We added a fifth limitation to discuss the implications of the low uptake of our intervention:*

“Fifth, the low uptake of VHW refill in the intervention arm may have underestimated the potential intervention effect. However, in the spirit of DSD it was important to offer a preference-based approach over both refill options simultaneously and analyse as such in an intention-to-treat analysis to assess the real-life effect if the model would be scaled up. The per protocol analysis including only the participants who opted for the VHW versus the control arm does not suggest better outcomes (S1 Table).”

2) The ~60% in the intervention who did not take up the offer of community-based ART care are important - who were they? what happened to them, did they access the standard of care instead and how did they adhere then? If they fell out of care completely then the solution to improving ART care needs to be sought elsewhere.

***Response:*** *The idea of the intervention was not that all participants opt for VHW follow-up. In the spirit of differentiated service delivery, the VIBRA package aimed at offering a preference-based approach where participants could choose between clinic-based care and VHW-based care. We assessed in more detail the flow of participants choosing and not choosing VHW follow-up in the intervention arm. We added the flow as an additional figure in the supplement (S2 Figure) and present it in the last paragraph of the Results section:*

“In the intervention arm, 48 and 70 opted for VHW or clinic follow-up, respectively. Among the participants who opted for drug refill by the VHW, 14 (29%) never linked to care (VHW or clinic), whereas 27 of the 70 (39%) who opted for clinic refill never linked to care. At 12 months follow-up, 12/48 (25%) were still followed by the VHW, 14/48 (44%) had transferred to the clinic and 15/48 (31%) were not in care. Among those who had opted for clinic refill, 38/70 (54%) were in care at 12 months (S2 Figure).”

*We also looked at baseline characteristics in those who chose VHW versus those who opted for the clinic in the intervention clusters:*

|  | **Total** | **Chose VHW** | **Chose Clinic** | **p-value*** |
| --- | --- | --- | --- | --- |
|  | **N=118** | **N=48** | **N=70** |  |
| Sex |  |  |  | 0.30 |
| Female | 67 (57%) | 30 (62%) | 37 (53%) |  |
| Male | 51 (43%) | 18 (38%) | 33 (47%) |  |
| **Age in years** | **36 (30-49)** | **40 (33-54)** | **34 (28-45)** | **0.006** |
| Regular sexual partner |  |  |  | 0.60 |
| Yes, one | 69 (58%) | 26 (54%) | 43 (61%) |  |
| Yes, several | 10 (8%) | 6 (12%) | 4 (6%) |  |
| No | 37 (31%) | 15 (31%) | 22 (31%) |  |
| Refused to answer or not applicable | 2 (2%) | 1 (2%) | 1 (1%) |  |
| HIV status of current partner |  |  |  | 0.51 |
| I don’t know | 63 (53%) | 24 (50%) | 39 (56%) |  |
| He/she is HIV-positive and on ART | 11 (9%) | 5 (10%) | 6 (9%) |  |
| He/she is HIV-positive but not on ART | 1 (1%) | 1 (2%) | 0 (0%) |  |
| He/she is HIV-positive but don’t know if he/she is on ART | 1 (1%) | 1 (2%) | 0 (0%) |  |
| I know that he/she recently tested negative | 3 (3%) | 1 (2%) | 2 (3%) |  |
| Missing | 39 (33%) | 16 (33%) | 23 (33%) |  |
| District |  |  |  | 0.061 |
| Butha-Buthe | 8 (7%) | 6 (12%) | 2 (3%) |  |
| Mokhotlong | 110 (93%) | 42 (88%) | 68 (97%) |  |
| HIV/ART history |  |  |  | 0.51 |
| Newly diagnosed | 92 (78%) | 35 (73%) | 57 (81%) |  |
| Known HIV+/never on ART | 11 (9%) | 5 (10%) | 6 (9%) |  |
| Previously on ART (stopped >30 days) | 15 (13%) | 8 (17%) | 7 (10%) |  |
| 7 years or more of schooling |  |  |  | 0.42 |
| No | 71 (60%) | 31 (65%) | 40 (57%) |  |
| Yes | 47 (40%) | 17 (35%) | 30 (43%) |  |
| Education |  |  |  | 0.14 |
| Secondary or higher education | 25 (21%) | 7 (15%) | 18 (26%) |  |
| No schooling or only primary | 92 (78%) | 41 (85%) | 51 (73%) |  |
| Missing | 1 (1%) | 0 (0%) | 1 (1%) |  |
| Employment |  |  |  | 0.96 |
| No employment and no regular income | 108 (92%) | 44 (92%) | 64 (91%) |  |
| (Self-)Employment with regular income | 10 (8%) | 4 (8%) | 6 (9%) |  |
| Alcohol abuse |  |  |  | 0.87 |
| No | 22 (19%) | 13 (27%) | 9 (13%) |  |
| Yes | 8 (7%) | 5 (10%) | 3 (4%) |  |
| n/a (no alcohol consumption) | 88 (75%) | 30 (62%) | 58 (83%) |  |
| History of local cannabis use |  |  |  | 0.12 |
| No cannabis use | 101 (86%) | 44 (92%) | 57 (81%) |  |
| Cannabis use | 17 (14%) | 4 (8%) | 13 (19%) |  |
| Main transportation to health facility |  |  |  | 0.83 |
| Taxi | 43 (36%) | 16 (33%) | 27 (39%) |  |
| Walk | 73 (62%) | 31 (65%) | 42 (60%) |  |
| Other | 2 (2%) | 1 (2%) | 1 (1%) |  |
| Round-trip travel cost (Maloti), median (IQR) | 0 (0-40) | 0 (0-50) | 0 (0-30) | 0.66 |
| Round-trip travel time (minutes), median (IQR) | 135 (80-360) | 220 (100-360) | 120 (60-360) | 0.13 |
| **Same-day ART prescribed?** |  |  |  | **0.001** |
| No | 25 (21%) | 3 (6%) | 22 (31%) |  |
| Yes | 93 (79%) | 45 (94%) | 48 (69%) |  |
| WHO stage |  |  |  | 0.22 |
| 1 | 116 (98%) | 46 (96%) | 70 (100%) |  |
| 3 | 1 (1%) | 1 (2%) | 0 (0%) |  |
| Missing | 1 (1%) | 1 (2%) | 0 (0%) |  |
| History of TB in the past |  |  |  | 0.35 |
| Yes | 5 (4%) | 3 (6%) | 2 (3%) |  |
| No | 110 (93%) | 43 (90%) | 67 (96%) |  |
| Missing | 3 (3%) | 2 (4%) | 1 (1%) |  |
| Presumptive TB |  |  |  | 0.24 |
| No | 98 (83%) | 37 (77%) | 61 (87%) |  |
| Yes | 17 (14%) | 9 (19%) | 8 (11%) |  |
| Missing | 3 (3%) | 2 (4%) | 1 (1%) |  |
| CD4 count, median (IQR) | 411 (254-526) | 390 (248-503) | 446 (255-578) | 0.32 |
| CD4 count groups |  |  |  | 0.69 |
| <200 | 19 (16%) | 11 (23%) | 8 (11%) |  |
| 200-499 | 36 (31%) | 21 (44%) | 15 (21%) |  |
| >500 | 27 (23%) | 13 (27%) | 14 (20%) |  |
| Missing | 36 (31%) | 3 (6%) | 33 (47%) |  |
| *Pearson’s chi2 or Fishers’ exact, where appropriate |  |  |  |  |

*Intervention participants that declined VHW follow-up were younger and more often also declined the offer of same-day ART initiation. We added this finding in the last paragraph of the Results section:*

“Comparing the baseline characteristics of intervention participants opting for VHWs with those opting for the clinic revealed that participants that opted for the clinic refill were younger (median 34 years vs 40 years) and more often also declined the offer of same-day ART initiation (31% vs 6%).”

*We suggest, however, to just add this statement and not to add the table above in the manuscript. First, as this was not a planned analysis and second, as the supplement already contains a lot of additional information already.*

*In the revised version we now discuss this aspect in Discussion section:*

“The participants who opted for clinic refill were younger (median 34 years) than those opting for the VHW. While it was important to offer a DSD model with both refill options available to choose from, this finding is important when decentralizing HIV care to lay health workers.”

3) I share Reviewer 2's concerns re regression model used in analysis, and the small numbers involved. The small numbers preclude drawing of reliable conclusions - only 41/188 participants opted for VHW-based refill, with unbalanced drop-out between the arms. Was there selection bias?
***Response:*** *The primary analysis included all individuals as randomized (intent-to-treat) to prevent introducing selection bias after randomization. In addition, all eligible individuals consented to participate. Please see our response to reviewer 2 for a further discussion regarding the regression model.*

4) Table 2 shows that engagement in care at 12 months (measured by VL assessment) was higher in the routine care arm than in the intervention arm, with an OR of 0.6 and a P-value of 0.058, which is borderline significant, and begs the question of whether the intervention would have been detrimental.

***Response:*** *We added a word of caution in the summary of the discussion (first paragraph):*

“Moreover, we found low overall uptake of the VHW refill option, a tendency to lower 12-month engagement in care and a numerically higher mortality in the intervention clusters.”

5) The mortality difference between arms is worrying, as is the SAE difference - both worse in the intervention arm (I is not clear to me that the causal relationship in the deaths is adequate - most of it is unknown, and could well have been associated with not being in regular care).

***Response:*** *According to Supplement Table 3 (S3 Table), of the 7 participants that died, 4 chose the VHW at enrolment. One never linked to the VHW or any other care despite several tracing attempts. Two linked to the VHW, clinically deteriorated, were referred timely to the health facility but died at the health facility or shortly after discharge. The fourth participant linked to the VHW, was asymptomatic during regular refills at the VHWs and died of a sudden death at home, suggestive for a sudden cardiac event or a stroke.*

*Overall, the detailed follow-up of the SAEs did not reveal a clear mismanagement by the VHW as cause of any of the SAEs. We clarified the findings in the Discussion section (fifth paragraph):*

“Even though similar mortality rates have been reported among newly initiated patients followed up in the community (26) and the cause of death is not clearly linked to a mis-management of any VHW (S3 Table), the imbalance of mortality between the groups warrants close monitoring when decentralizing HIV care.”

6) Reviewer 2 asks for a per-protocol analysis, and I agree that that would be of interest. It is currently not clear what happened with those in the intervention arm who declined VHW-based refill. Were they more or less likely to be VL-suppressed at 12 months, were the 7 deaths in the intervention group in this 60%, or in the 40% who accepted VHW-refills?

***Response:*** *We assessed in more detail the flow of participants choosing and not choosing VHW follow-up in the intervention arm. We added the flow as an additional figure in the supplement (S2 Figure) and present it in the last paragraph of the Results section. Moreover, we modified our per protocol analysis of the role of choosing the VHW, see last paragraph in Statistical analysis and Supplement Table 1 (S1 Table), comparing only the participants who opted for the VHW (n=48) versus the control arm (n=139). It does not suggest different outcomes (S1 Table). Regarding the question of the 7 deaths of the intervention group, we refer to the answer of the previous comment.*

***** Comments from the reviewers:**

***** Reviewer #1:**

*Statistical review*

This paper reports a cluster randomised trial investigating an intervention using village based lay workers to initiate ART amongst individuals who are HIV positive. The trial appears to have been well-conducted, with the results not showing an advantage of the intervention. I have some minor comments, listed below.

1) Abstract: I'd recommend the secondary outcomes are reported in a similar manner to the primary outcome.

***Response:*** *We adapted accordingly.*

2) Line 174: can the authors say more about the approach used to stratify the randomisation?

***Response:*** *Randomisation was stratified by district (Butha-Buthe versus Mokhotlong), village size (≥30 versus <30 households) and access to the nearest health facility (easy versus hard to reach, defined as needing to cross a mountain or river or >10 km away from a health facility). The stratification data was obtained for all 648 eligible villages on the initial list, i.e. before the random cluster selection and randomization. The study team double-checked the data on the list with the coordinators of all participating health facilities. This process is being described in section “Cluster sampling and randomization” and in more detail in the published study protocol of VIBRA and HOSENG trial. We added a reference to the study protocols as a last sentence in that paragraph:*

“We describe the process of the cluster selection and randomisation in detail in the two published study protocols of HOSENG(10) and VIBRA trial(9).”

3) Line 250: I'm not sure it is clear in the statistical analysis section how dried blood spot samples that were below limit of detection were treated for the original viral suppression endpoint of <20. Were they treated as non-suppressed (as implied in line 280)?

***Response****: That’s correct. We clarified in the Outcomes section:*

“Over the course of the study, clinics were increasingly collecting blood samples for VL measurement using dried blood spot, instead of plasma, with a lower limit of detection of 400 copies/mL and would thus be classified as unsuppressed for the pre-specified primary endpoint.”

4) Results/table 3 - it would be useful for future research to report the ICC for the primary endpoint.

***Response:*** *The ICC for the primary endpoint is 0.01. This has now been added to the manuscript, at the end of the second paragraph of the Results section.*

5) Results - I would recommend a safety paragraph that reports any adverse events and the 7 deaths that occurred. Were these deaths linked to the intervention at all? 7 in one arm and 0 in the other is, I think, statistically significant. Mortality is listed as a secondary outcome, but the authors appear to not analyse it as one.

***Response:*** *Formally, the difference between 7/118 versus 0/139 deaths is significant (Fisher’s exact). The follow-up of the SAEs revealed that 3 of the 7 participants with SAE chose the clinic, not the VHW, as place of refill, and a clear mismanagement of the VHW for the other 4 could not be established (see detailed answer of comment number 5 of the academic editor). Thus, we suggest formulating these findings cautiously and not to report a p-value. However, we added a word of caution in the Discussion section (first paragraph):*

“Even though similar mortality rates have been reported among newly initiated patients followed up in the community (26) and the cause of death is not clearly linked to a mis-management of any VHW (S3 Table), the imbalance of mortality between the groups warrants close monitoring when decentralizing HIV care.”

6) I'd recommend a CONSORT checklist (extension to cluster randomised trials) is provided.

***Response:*** *We submitted a CONSORT checklist and referred to it in the last paragraph of “Study design and participants”*

***** Reviewer #2:**

Overall
This study describes the result of a cluster randomized trial of an offer of community-based ART refill after home based initiation compared to clinic based refill. The question is of importance and the study appears to have been well executed. Still there are some concerns about the methods that would need to be addressed before this was suitable for publication.

Major Points

1) While the sample size is appropriate from a power standpoint, the cluster randomized design with small(ish) numbers leaves concerns about the comparability of the results.  There are differences between populations at baseline (not dramatic, but they exist) and as such, were any of the baseline variables specified a priori as variables that should be controlled in the final model? It does not appear most of what is somewhat imbalanced in Table 1 is adjusted for in Table 2.

***Response:*** *A cluster randomized design was chosen as the intervention was delivered at the cluster level and contamination would have been a genuine concern if the intervention was individually randomized. At the same time, as those eligible for the study must be HIV-positive and not on ART, this meant that the number of potentially eligible individuals was small (median = 3, IQR 2-5, range 1-11). We did not specify any variables to adjust for apriori, but rather specified in the protocol and the statistical analysis plan that Table 1 was to be reviewed by the team prior to looking at any outcome data in order to decide if any variables should be adjusted in the final model. This was done and it was decided that there was not sufficient and clinically significant imbalance in any baseline variable and the sample size too small to realistically adjust for them.*

2) The primary endpoint was defined as being virally suppressed an IN CARE. It isn't clear how this was defined since the home-based refill arm (for those who chose it) didn't go to the clinic. Did the definition differ by arm?

***Response:*** *Blood draw for viral load happened at the clinics, also for those participants who chose the VHW. In care was defined as at least one clinic visit in the outcome window (mentioned in the second paragraph of the Outcomes section). Thus, having a viral load measurement in the primary endpoint window was considered as ‘in care’ and the same definition applied in both arms. We clarified in the Outcomes section as follows:*

“Blood draw for viral load for all study participants was conducted at the clinics and the analysis was performed at the corresponding laboratories of the study districts using COBAS TaqMan® HIV-1 Test, v2.0, Roche Diagnostics.”

3) The most interesting finding in this study is that in the intervention arm, less than half wanted home-based refill. The challenge is in figuring out what this generalizes to. The sample size is fairly small and somewhat selected (though not overly so) making it a challenge to determine who this generalizes to. It would help if the authors would discuss this more.

***Response:*** *We looked at the intervention participants in more detail (see answer to comment 2 of the academic editor), e.g. the group of intervention participants that opted for clinic refill was significantly younger (median 34 years) than those who opted for the VHW. This is important to take into account when offering the model, but does not necessarily limit generalizability. We added a few more thoughts about this aspect in the Discussion section:*

1) *Second paragraph of Discussion section:*

“The participants who opted for clinic refill were younger (median 34 years) than those opting for the VHW. While it was important to offer a DSD model with both refill options available to choose from and to analyze as such in an intention-to-treat analysis, this finding is important when decentralizing HIV care to lay health workers.”

2) *Fifth limitation in Discussion section:*

“Fifth, the low uptake of VHW refill in the intervention arm may have underestimated the potential intervention effect. However, in the spirit of DSD it was important to offer a preference-based approach over both refill options simultaneously and analyse as such in an intention-to-treat analysis to assess the real-life effect if the model would be scaled up. The per protocol analysis including only the participants who opted for the VHW versus the control arm does not suggest better outcomes (S1 Table).”

4) The main results tell us that just under half of the participants had a suppressed viral load at follow up, but we need to know how many had a viral load.

***Response:*** *Thank you for pointing out this omission. We added a footnote to table 2 to clarify how many participants had a missing VL measurement in the primary endpoint window:*

“[4] 23/257 (9%), 14 in control and 9 in intervention, did not have a viral load measurement in the primary endpoint window; these were considered to be failures.”

5) The authors comment that the differences between arms are not significant. This is true but the sample size is small, and the study was powered on 20% differences. Surely a 10% reduction in viral suppression would be seen as a strong negative against home based refills and here we see a 7% difference. So I would not consider these result to be equivalent.

***Response:*** *We adapted the summary of the discussion (first paragraph) to clarify that some results (esp. the difference in engagement in care at 12 months) may point towards a detrimental outcome of the intervention:*

“Moreover, we found low overall uptake of the VHW refill option, a tendency to lower 12-month engagement in care and a numerically higher mortality in the intervention clusters.”

6) I'm not clear on why no formal comparison (RD, 95% CI) is provided for mortality. The differences are concerning.

***Response:*** *We kindly refer to the answer of comment 5 of Reviewer #1.*

7) Given your outcomes are common, use of logistic regression is exaggerating differences between groups in your relative comparisons. Relative risk models (log binomial) would be more appropriate here.

***Response:*** *Thank you for your comment and suggestion. We have added the risk ratio to Table 2 and Supplemented Table S1.*

8) While the sample size is small, I would still like to see some kind of per protocol analysis that assessed the effect of actually taking up home based refill. This is challenging to do because it was an offer not a requirement and the numbers uptaking the offer reduce the sample size substantially but even if the results end up very imprecise, it would be helpful to know if those who chose home based refills had better results (adjusted appropriately) than those who did not.

***Response:*** *Thank you for this important comment. We kindly refer to the answer of comment 6 of the academic editor.*

Minor points

9) In the abstract, for consistency, put the order of results the same for each (intervention then control).

***Response:*** *We adapted accordingly.*

10) We are told that in the intervention arm, 41% opted for home-based refill. Can you provide a confidence interval for this finding?

***Response:*** *The 95% confidence interval is 32 – 50%. We added the confidence interval to the Results section, second paragraph.*

***** Reviewer #3:**

In the study investigators examine the effect of offering home based into retroviral therapy refills after home-based therapy initiation in Lesotho as compared to home-based therapy initiation with facility-based follow up. Overall, 157 adults were randomized and no differences were found in viral suppression after six or 12 months.

While the notion of follow up in the community is important, I think there are several problems with this trial that attenuate its utility.

1) First, there is not enough justification provided to motivate the home based follow up.  In other words, the choice of an intervention must be well justified for a study to be compelling.  I am not saying there is no rationale, but the authors have not provided a clear rationale.  Is it based purely on distance? Is there some element of the clinic that is unwelcoming and intimidating? Is this all about the opportunity costs? Can the authors say more about the rationale for follow up at home? In particular given the fact that structural barriers were not commonly reported in the previous work. In other words, if the justification for follow-up at home was due to stigma and or distrust at the healthcare facility then a village health worker-based approach would be in theory useful. But the others have not provided an underlying theoretical reason. The need for a theoretical or evidence-based justification comes to a head because the majority of patients randomized to community follow up did not opt for village health worker follow up because they felt like they did not trust the village health worker. This represents a problem for the study for several reasons.  First, if the hypothesis is about location of services, and the real barrier is the cadre, then the study has tested the wrong intervention.  Furthermore, it basically seems that they offered an intervention that was not needed in this setting. But that does not mean the study provides evidence that home based follow up is not useful or effective, which is how this study will be interpreted. So, consider a scenario in which patients did in fact trust the community health worker. In the case the intervention of home-based delivery could have worked. So, the fact that there were no effects in this trial is not an indictment of the notion of home-based treatment but rather of the way in which the relationship was created. In other words, the main scientific issue is really the nature of the relationship with the health care worker rather than the location of services.  This comes back to the justification for the trial intervention.  The absence of a justification makes the fact that people didn't want what was offered.

If this boils down to the fact that people didn't want to follow up in the community with VHW, it seems like that could have been known with adequate formative work and therefore avoided the conduct of an expensive trial.

***Response:*** *Thank you for this important comment. We kindly refer to the answer of comment 1 of the academic editor.*

3) The paper is also thin on mediation and how and why trust was low. Other implementation outcomes would be useful - acceptability.  Was the issue trust of the VHW in general or trust of any health professional coming to the home? Was it trust about disclosure or trust that the VHW was competent?  Do CHW provide other services and do people trust those?  Such details would help create some contextual understanding of the results, perhaps using some mixed methods.
***Response:*** *We collected the reasons for refusing the VHW refill at the day of enrolment by the study nurses that also enrolled the participants. However, due to limited time, the study nurses were not able to collect in-depth data on the underlying causes of e.g. the mistrust towards the VHW. Further qualitative research is needed. We added this important point as a sixth limitation of our study:*

“Sixth, due to logistical reasons, we were not able to collect in-depth data on the reasons for refusing the VHW refill at enrolment, for example the underlying cause of mistrust. Further qualitative research is warranted.”
