# Supplementary material for: Offering ART refill through community health workers versus clinic-based follow-up after home-based same-day ART initiation in rural Lesotho: The VIBRA cluster-randomized clinical trial
Source: PLoS Med. 2021 Oct 21;18(10):e1003839. doi: 10.1371/journal.pmed.1003839 (PMC8568187; doi:10.1371/journal.pmed.1003839)
Supplement: Attachment — Submitted filename: VIBRA_response_to_reviewers_20210927.docx [file pmed.1003839.s007.docx]

**Response to Reviewers**

*Offering ART refill through community health workers versus clinic-based follow-up after home-based same-day ART initiation in rural Lesotho: The VIBRA cluster-randomised clinical trial*

***** Requests from the editors:**

1) In the abstract, please add "The trial was registered ..." or similar prior to the registration number.

***Response:*** *We adapted accordingly.*

2) Please make that "... in the intervention arm ..." and similar in the abstract.

***Response:*** *We adapted accordingly.*

3) Does "WVH" need to be corrected late in the abstract?

***Response:*** *Thank you for spotting this error. We adapted accordingly.*

4) Noting reference 11 and others, please ensure that all references have full access details.

***Response:*** *We adapted accordingly.*

5) Please remove all iterations of "[Internet]" from the reference list.

***Response:*** *We adapted accordingly.*

***** Comments from reviewer #1:**

1) Thank you to the authors for addressing my previous comments well. I have no further issues to raise.

***Response:*** *Thank you very much.*

***** Comments from reviewer #2:**

1) I am happy with all the edits made except the lack of a formal comparison of the deaths. The authors note that this is a small study and use this as justification for not focusing on differences in baseline characteristics between the arms. This is a reasonable response since, even though I would prefer adjustment for some differences (like CD4 count) the random error summar does account for these. But the fact that you did find important differences in mortality is very concerning and needs to include a formal comparison.

***Response:*** *We understand the reviewers’ concern. We added the Fisher’s exact (p-value of 0.0039) to the manuscript. However, as the deaths do not appear to be linked to a mismanagement of any of the VHWs, we have to be careful how to interpret these findings and also added more description to the Results section:*

“Seven (6%) serious adverse events, all deaths, occurred in the intervention as compared to zero (0%) in the control arm (p=0.0039). Of the 7 participants who died, only 4 chose the VHW at enrolment. Out of these, one never linked to the VHW or any other care despite several tracing attempts. Two linked to the VHW, clinically deteriorated, were referred timely to the health facility but died at the health facility or shortly after discharge. The fourth participant linked to the VHW, was asymptomatic during regular refills at the VHW and died of a sudden death at home, suggestive for a sudden cardiac event or a stroke. Overall, the detailed follow-up of the SAEs did not reveal a clear mismanagement by the VHWs as cause of any of the SAEs (S3 Table).”

***** Comments from reviewer #3:**

1) I am satisfied with the reviewer responses. My concern that the trial could be much more informative if we knew more about the reasons for mistrust appears to be something that cannot be addressed. I would encourage some more discussion of this issue, in particular a call out to future researchers to take the issue of patient preferences seriously and position themselves to measure such perspectives especially when the intervention depends on them.

***Response:*** *Thank you. We agree with the reviewer and added some more clarity to that limitation in the Discussion section:*

“Nevertheless, more formative piloting work would have been beneficial and more research to assess the clients’ preferences for ART refill in rural Lesotho is needed.”
