# Supplementary material for: Offering ART refill through community health workers versus clinic-based follow-up after home-based same-day ART initiation in rural Lesotho: The VIBRA cluster-randomized clinical trial
Source: PLoS Med. 2021 Oct 21;18(10):e1003839. doi: 10.1371/journal.pmed.1003839 (PMC8568187; doi:10.1371/journal.pmed.1003839)
Supplement: Attachment — Submitted filename: VIBRA_response_to_reviewers_20211002.docx [file pmed.1003839.s008.docx]

**Response to Reviewers**

*Offering ART refill through community health workers versus clinic-based follow-up after home-based same-day ART initiation in rural Lesotho: The VIBRA cluster-randomised clinical trial*

***** Requests from the editors:**

Please finalize the arrangements for data access and detail these in the submission form. 
***Response:*** *We have finalized our data repository on Zenodo. It can be accessed under the following doi link:* **https://doi.org/10.5281/zenodo.5547573***. The doi link was added to the data access statement.*

At line 60 (abstract), please mention the setting, as early in the Methods section (main text). 
***Response:*** *We adapted accordingly.*

At line 70, please avoid beginning the sentence with "48/118" and adapt the sentence to "In the intervention arm, 48/118 ..." or similar.
***Response:*** *We adapted accordingly.*

At line 72, should that be "adjusted risk difference"?
***Response:*** *Yes, thank you. We adapted accordingly.*

At line 73, please adapt the text to "... engagement in care was non-significantly lower in the intervention arm (adjusted difference ...". Please make a similar amendment at line 103.
***Response:*** *We adapted accordingly.*
At lines 75 and 104, please make that "... intervention and control arms, respectively.".
***Response:*** *We adapted accordingly.*

At lines 84, 111 and 526, please remove "rather".
***Response:*** *We adapted accordingly.*

At line 102, we suggest removing the p value and instead stating "... with no significant difference between arms." or similar.
***Response:*** *We adapted accordingly.*

Please remove the trade mark at line 283, and any other instances throughout the paper. 
***Response:*** *We adapted accordingly.*

We believe that the trial registration number is quoted twice in the Methods section (main text), and once will suffice. 
***Response:*** *Thank you. We removed the one right before the Results section.*

At line 437, please remove "the" preceding "UNAIDS".
***Response:*** *We adapted accordingly.*

For references 1 & 16, please specify a specific web page and add an accessed date. 
***Response:*** *We adapted accordingly.*

Noting reference 5, please use the journal name abbreviation "PLoS Med." consistently. 
***Response:*** *We adapted accordingly.*

Noting reference 11, please ensure that all references have full access details. 
***Response:*** *We adapted accordingly.*

In other references, please use the journal name abbreviations "PLoS ONE" and "JAMA".

***Response:*** *We adapted accordingly.*

We suggest moving S1 figure to the main body of the paper. 
***Response:*** *We adapted accordingly.*


***** Comments from reviewer #2:**

Thank you for the revisions based on my comment. I appreciate the addition of the formal comparison. My personal feeling is that last sentence added is unnecessary as it make a case that this was not the VHW's fault, but this is not the point of the study, the point is whether the overall intervention is safe. We can't conclude that just because your evaluations led you to conclude that the VHW was not responsible doesn't mean that there is not increased risk associated with the intervention. That said, I don't see that what you have said is wrong, so I will leave it to the editors to decide if they want any additional changes based on this point.
***Response:*** *Thank you. We removed the last sentence (line 433).*
